# Intentional polarity conversion of AlN epitaxial layers by oxygen

**DOI:** 10.1038/s41598-018-32489-w

**Published:** 2018-09-20

**Authors:** N. Stolyarchuk, T. Markurt, A. Courville, K. March, J. Zúñiga-Pérez, P. Vennéguès, M. Albrecht

**Affiliations:** 1 Leibniz-Institut für Kristallzüchtung, Max-Born-Straße 2, 12489 Berlin, Germany; 2 Université Côte d’Azur, Centre de Recherche sur l’Hétéro-Epitaxie et ses Applications, Rue Bernard Grégory, Sophia Antipolis, 06560 Valbonne, France; 3 Laboratoire de Physique des Solides, Université Paris-Sud, CNRS-UMR 8502, 91405 Orsay, France

## Abstract

Nitride materials (AlN, GaN, InN and their alloys) are commonly used in optoelectronics, high-power and high-frequency electronics. Polarity is the essential characteristic of these materials: when grown along c-direction, the films may exhibit either N- or metal-polar surface, which strongly influences their physical properties. The possibility to manipulate the polarity during growth allows to establish unique polarity in nitride thin films and nanowires for existing applications but also opens up new opportunities for device applications, e.g., in non-linear optics. In this work, we show that the polarity of an AlN film can intentionally be inverted by applying an oxygen plasma. We anneal an initially mixed-polar AlN film, grown on sapphire substrate by metal-organic vapor phase epitaxy (MOVPE), with an oxygen plasma in a molecular beam epitaxy (MBE) chamber; then, back in MOVPE, we deposit a 200 nm thick AlN film on top of the oxygen-treated surface. Analysis by high-resolution probe-corrected scanning transmission electron microscopy (STEM) imaging and electron energy-loss spectroscopy (EELS) evidences a switch of the N-polar domains to metal polarity. The polarity inversion is mediated through the formation of a thin Al_x_O_y_N_z_ layer on the surface of the initial mixed polar film, induced by the oxygen annealing.

## Introduction

Group III-nitride materials (AlN, GaN, InN and their alloys) lack inversion symmetry along the c-direction, i.e., the films exhibit either an N-polar or a metal (Al, Ga, In) polar surface. The physical properties of such films, e.g., the polarization fields in strained heterostructures, the incorporation of impurities and surface diffusion critically depend on the polarity of the film^1–4^. Hence, control of polarity during growth on non-polar substrates is a significant issue in III-Nitride epitaxy.

A conventional approach to achieve metal-polar films in metalorganic vapor phase deposition includes nitridation of the sapphire substrate and deposition of a low-temperature buffer layer; without a buffer layer the film exhibits mixed polarity^5–8^. In the latter case, lateral overgrowth of either N- or metal-polar domains can be promoted to obtain a single polarity, by applying appropriate growth conditions^9–11^. Nanowires often grow mixed-polar (predominantly, N-polar), which causes differences in the incorporation of indium and, consequently, drastically affects the characteristics of the devices based on such structures^12–14^. In contrast to planar layers, lateral overgrowth is not an option to achieve homo-polar nanowires. Therefore, an alternative approach to intentionally convert polarities during growth is highly desirable. Such an approach would also open perspectives to realize epitaxial structures with intentionally induced periodic polarity inversion along the growth direction, which holds the promise to build quasi-phase-matched (QPM) structures for second harmonic generation (SHG)^15,16^. The wide extent of III-Nitrides (GaN, AlN, InN) bandgaps makes these materials particularly attractive for nonlinear optics applications since it allows to generate emission from the deep UV (for AlN) to far infra-red (for InN)^17^.

Other research groups already demonstrated intentional conversion from metal- to N-polarity by Mg exposure of the growth surface during both metalorganic vapor phase deposition (MOVPE) and molecular beam epitaxy (MBE). In this case, a zig-zag shaped inversion domain boundary, formed of Mg_3_N_2_, promotes the polarity conversion in GaN^18–21^. However, the opposite inversion of polarity – from N- to metal-polar GaN was not demonstrated with this approach.

In a previous work of our group, we have shown that the formation of Al_x_O_y_N_z_ is critical for switching N-to Al-polarity in III-Nitrides grown on sapphire^22,23^. The formation of Al_x_O_y_N_z_ in that case is due to a reaction between sapphire and ammonia during nitridation. We adopt these results and present the possibility to convert the polarity of N-polar AlN domains in a mixed-polar AlN epitaxial film to Al-polar using an oxygen plasma.

Our study, based on a detailed structural analysis of the resulting layer by high-resolution probe-corrected scanning transmission electron microscopy (STEM) imaging and electron energy-loss spectroscopy (EELS), shows a conversion of N-polar domains to metal polar domains through the formation of a thin Al_x_O_y_N_z_ layer on the surface of the mixed polar film as a result of the oxygen annealing. The atomic structure of the Al_x_O_y_N_z_ is in excellent agreement with the one that forms during nitridation of sapphire (0001) substrates in an MOVPE reactor^22^.

The studied sample is grown in two steps. In the first step, the AlN film approximately 280 nm thick is deposited at 1080 °C on the pre-nitridated sapphire surface. After this, the process is interrupted, and the sample is transferred into a plasma-assisted molecular beam epitaxy (MBE) chamber, where it is heated up to around 550 °C for 30 minutes under a flow of oxygen. After annealing in oxygen plasma, the sample is transferred back to the MOVPE growth reactor, where a 200 nm thick AlN film is grown on top of the oxygen-plasma treated sample surface at 1080 °C. Two samples – one where the growth was stopped after the first step (before oxygen annealing) and the second after oxygen annealing and AlN overgrowth – are analyzed by conventional transmission electron microscopy (TEM) and high-resolution high-angular annular dark-field STEM (HAADF-STEM). The latter sample is also analyzed by EELS.

## Results

**Figure 1:**
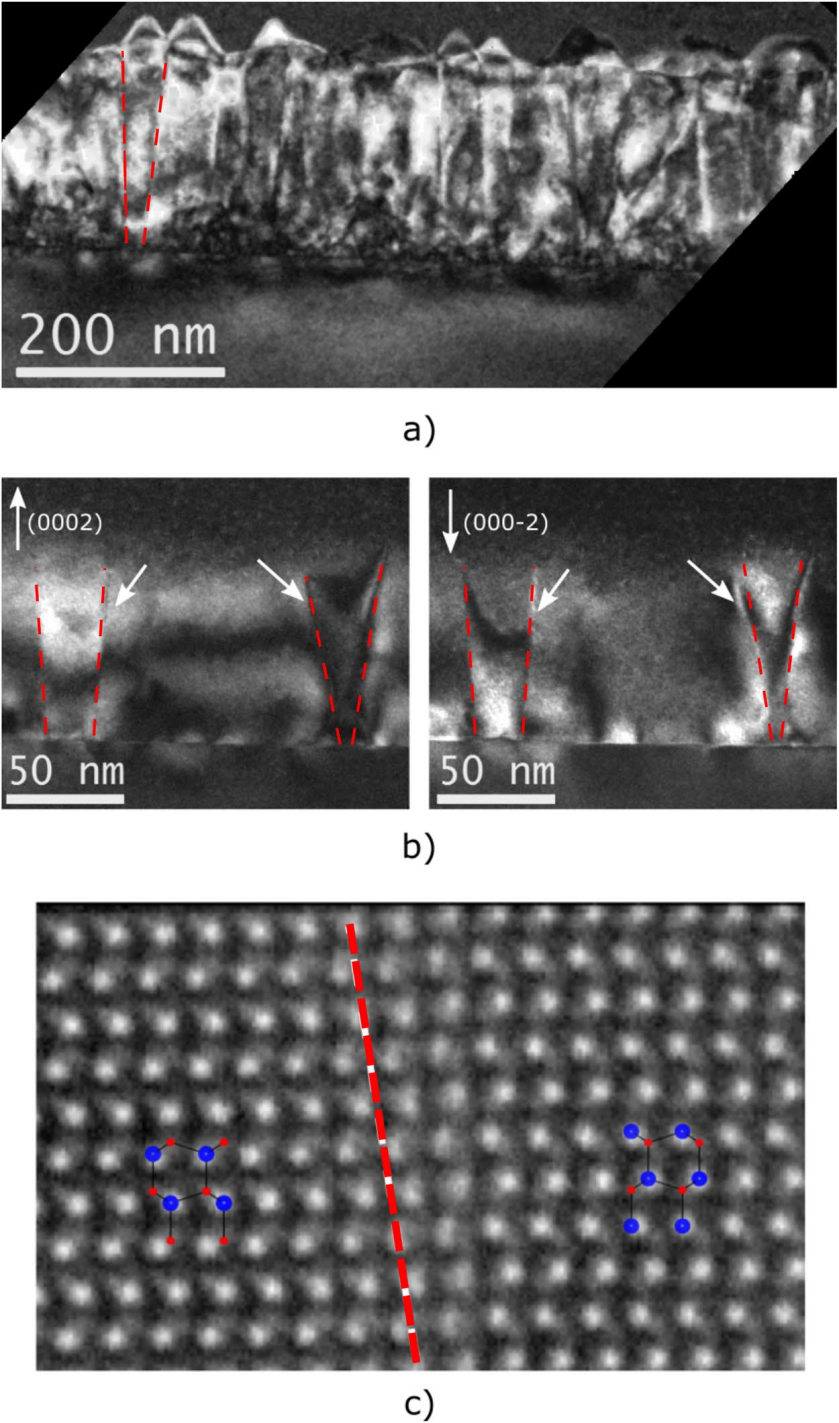
TEM analysis of AlN film deposited at a high temperature on a pre-nitridated sapphire substrate. (**a**) Cross-sectional many-beam dark-field image in [10–10]_AlN_ zone axis, revealing the presence of crystalline columns in AlN layer. (**b**) Dark-field images of columnar domains with g = (0002) and g = (000–2) in the [10–10]_AlN_ zone axis. White arrows indicate contrast reversal, characteristic for inversion domains. (**c**) High-resolution HAADF-STEM image in [11–20]_AlN_ zone axis of a boundary between two domains. Aluminum atomic columns appear with higher intensity, and nitrogen atomic columns with lower, due to the difference in atomic numbers Z. The AlN layer is N-polar on the left side of the boundary, marked by a dashed line, and Al-polar on the right side of the boundary. On the schematic models, blue balls depict Al atoms, and red – N atoms.

Figure 1 is a dark-field image (g = (0002)) of an AlN film after the first growth step in the [10–10] zone axis. It shows columns that are facetted on the surface (Fig. 1(a)). The columns are 15–30 nm wide at the base, and their side facets are inclined by 2–5° from the c-direction, thus resembling a V-shape. Figure 1(b) shows dark field images of the AlN film recorded in thinner part of the TEM specimen, taken with g = (0002) and g = (000–2) (b) in the [10–10] zone axis. The inversion of the contrast between the V-shaped columns and the surrounding matrix when switching from g_0002_ to g_000–2_ is characteristic for the presence of inversion domains in non-centrosymmetric crystals^24,25^. Figure 1(c) shows a magnified high-resolution HAADF-STEM image of a domain boundary between N-polar and Al-polar regions (inversion domain boundary). Aluminum atomic columns appear with higher intensity, and nitrogen atomic columns with lower intensity, due to the difference in their atomic number Z. The AlN layer is N-polar outside the V-shaped column (on the left side of the boundary, marked by the dashed line), and Al-polar inside it (on the right side of the boundary). Thus, the AlN layer before oxygen annealing is mixed-polar with an N-polar matrix and Al-polar columnar inversion domains.

**Figure 2:**
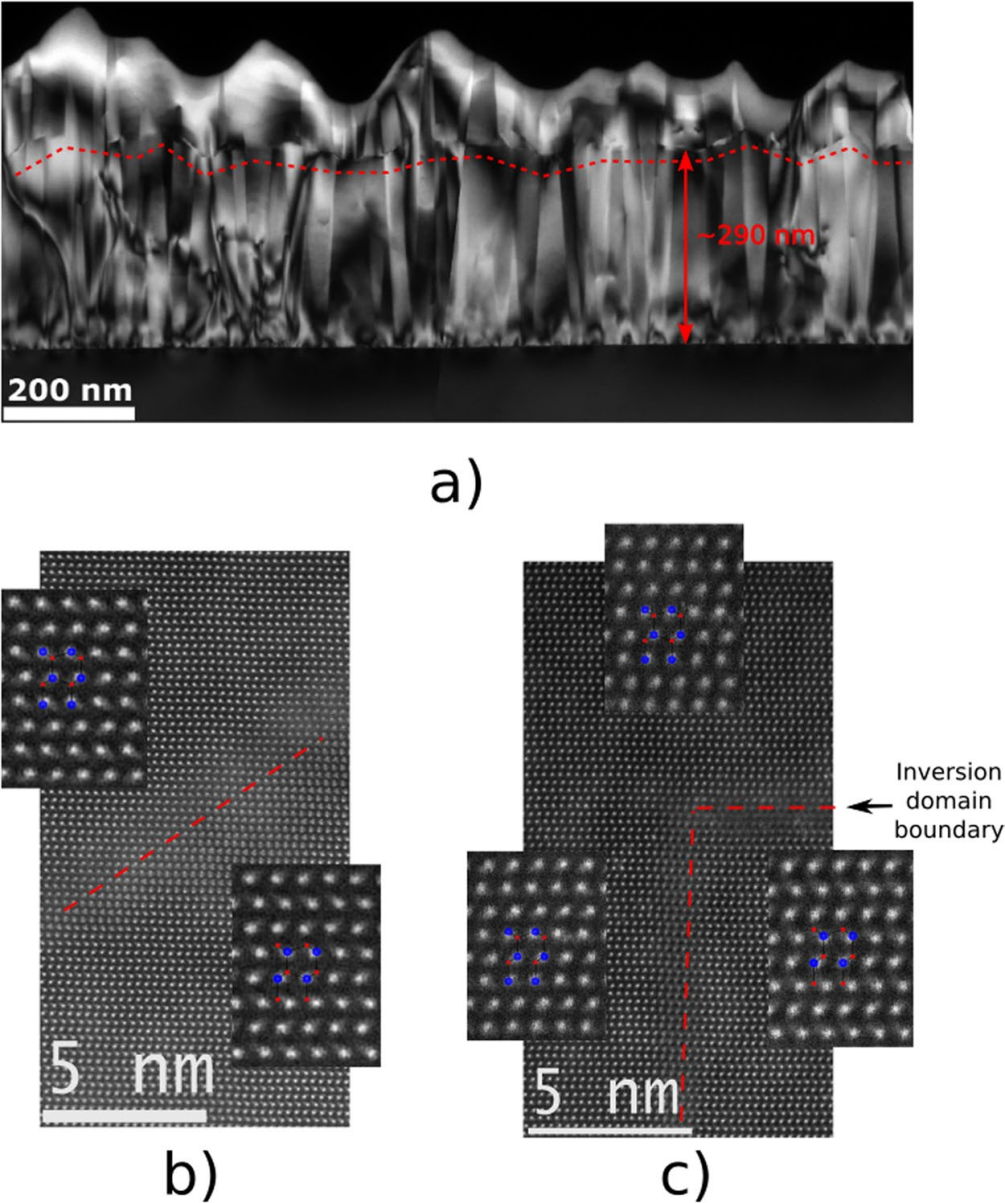
TEM analysis of AlN layer after treatment with oxygen plasma and AlN film regrowth. (**a**) Dark-field (g = (0002)) cross-section image close to the [11–20]_AlN_ zone axis. Red arrow denotes the thickness of AlN layer, deposited during the first step. The zig-zag boundary that divides the AlN layer deposited before and after the oxygen-annealing step is distinguishable; (**b** and **c**) High-resolution HAADF-STEM images of inclined boundary regions in [11–20]_AlN_ zone axis, revealing the layer polarity.

Figure 2 shows a dark-field (g = (0002)) cross-sectional TEM image close to the [11–20] zone axis of the AlN layer after treatment with oxygen plasma and overgrowth by an AlN film. The image reveals an irregular zig-zag shaped boundary (schematically indicated by a red line) 250–300 nm away from the interface with the sapphire substrate. Since this distance corresponds well to the thickness of the initially deposed AlN, it is natural to assume that this interface is linked to a structural change due to the oxygen plasma treatment during the growth interruption; thus, the layer above the boundary corresponds to the AlN film deposited in the second growth step. The irregular zig-zag shaped boundary does not exhibit any preferred plane but consists of varying inclined and horizontally aligned segments.

High-resolution HAADF-STEM imaging of an inclined boundary region in the [11–20] projection (Fig. 2(b)) reveals the polarity: below the boundary, the AlN layer is N-polar and above the boundary – Al-polar. The dashed line indicates the boundary between two regions with different polarities. Figure 2(c) shows an area of the initial AlN film where an Al-polar columnar inversion domain (on the left) adjoins the N-polar matrix (on the right). The horizontal dashed line denotes the boundary between the layers grown before and after oxygen annealing, and the upper layer is Al-polar. This means that the polarity of the N-polar matrix is inverted due to the oxygen annealing, while the polarity of Al-polar columns persists. A large number of additional HAADF-STEM measurements supports our observations that the layer above the boundary is predominantly Al-polar. From now on we will refer to the zig-zag boundary as “inversion domain boundary” (IDB) since it switches the polarity of N-polar regions.

**Figure 3:**
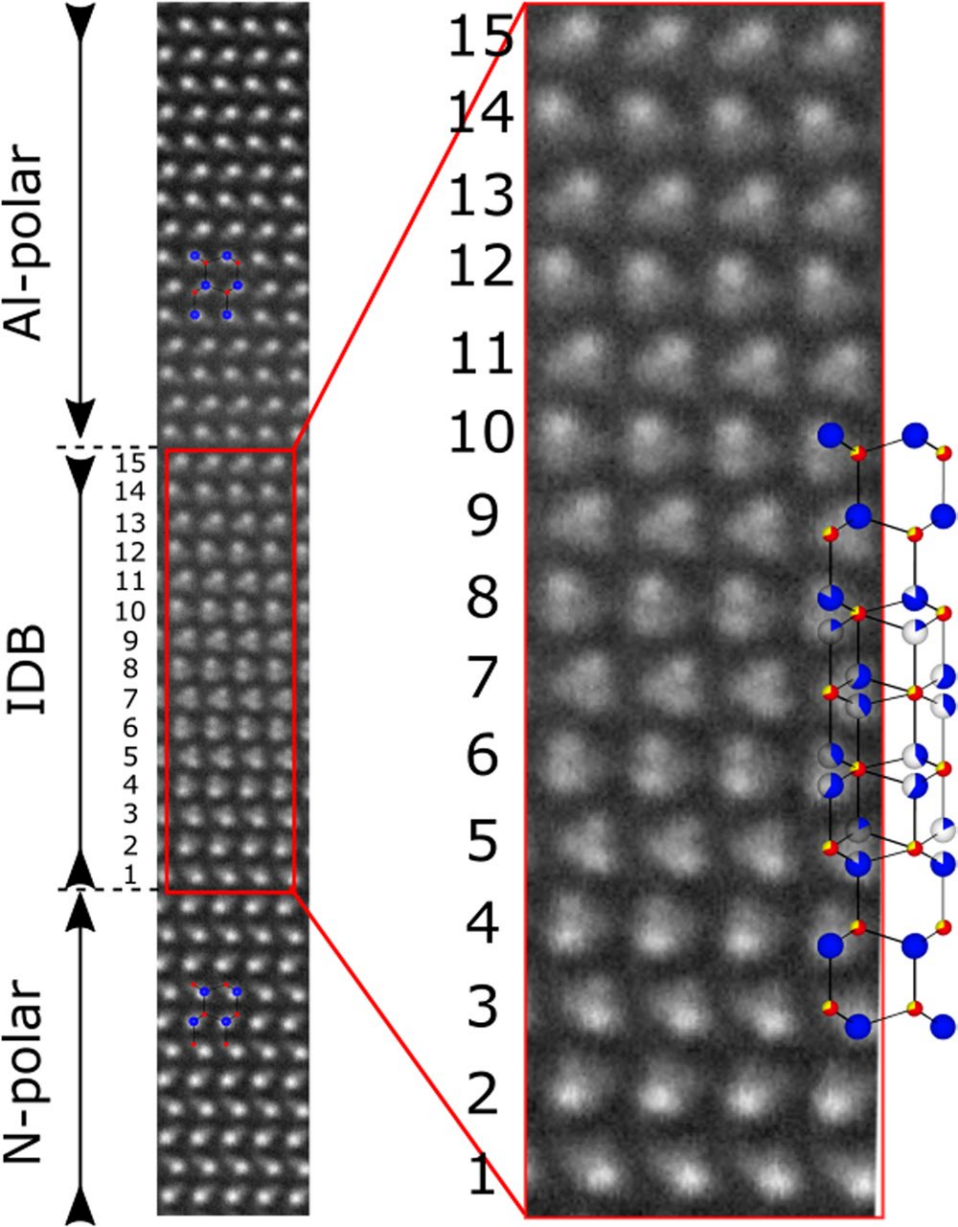
High-resolution HAADF-STEM image, revealing the microstructure of the inversion domain boundary between an N-polar domain of first AlN film and final Al-polar AlN layer. Theoretical stick-and-ball model of Al_9_O_3_N_7_ is shown for comparison. (The anion positions (small balls) are shared between N (red) and O (yellow) atoms. The cation positions (big blue balls) are occupied by Al and Al vacancies, thus displayed with different filling ratios).

In the magnified view of the IDB (Fig. 3), we observe a segment with 15 monolayers characterized by triangular contrast pattern, which separates two wurtzite lattices: the lattice below it is N-polar AlN, and above it – Al-polar. The triangular contrast pattern, where the polarity inversion occurs, corresponds well to the already proposed IDB model based on the polytypoid phase of Al_x_O_y_N_z_^26–28^. The IDB can be described by two interpenetrating N-polar and Al-polar wurtzite lattices sharing a common anion sub-lattice. Cations occupy both the upper (Al-polar lattice) and lower (N-polar lattice) symmetric tetrahedral sites. From the 1^st^ to 5^th^ monolayers (see magnified image in the red frame) the HAADF-intensity is higher at the lower cation site (“N-polar”); from the 6^th^ to 9^th^ monolayer it is comparable for both “N-polar” and “Al-polar” sites; from 10^th^ monolayer the intensity maximum shifts to the upper cation position (“Al-polar”). Since the HAADF-intensity of the cation columns is associated with the occupation of the respective sites, thus, within the IDB, the gradual change of the cation sites occupation, from N-polar to Al-polar sites establishes Al-polarity.

The stick-and-ball model of Al_9_O_3_N_7_ is shown along with the experimental image on Fig. 3; the anion sites in this model are statistically distributed between O and N atoms. The transition interface between N-polar and Al-polar wurtzite on the experimental image consists of 13–14 monolayers instead of 4 monolayers in the Al_9_O_3_N_7_ model by Asaka *et al*.^26^. We ascribe this to the three-dimensional irregular shape of the IDB resulting in a projection of the inclined facets of the IDB onto the image plane that apparently increases the width of the IDB in the image.

**Figure 4:**
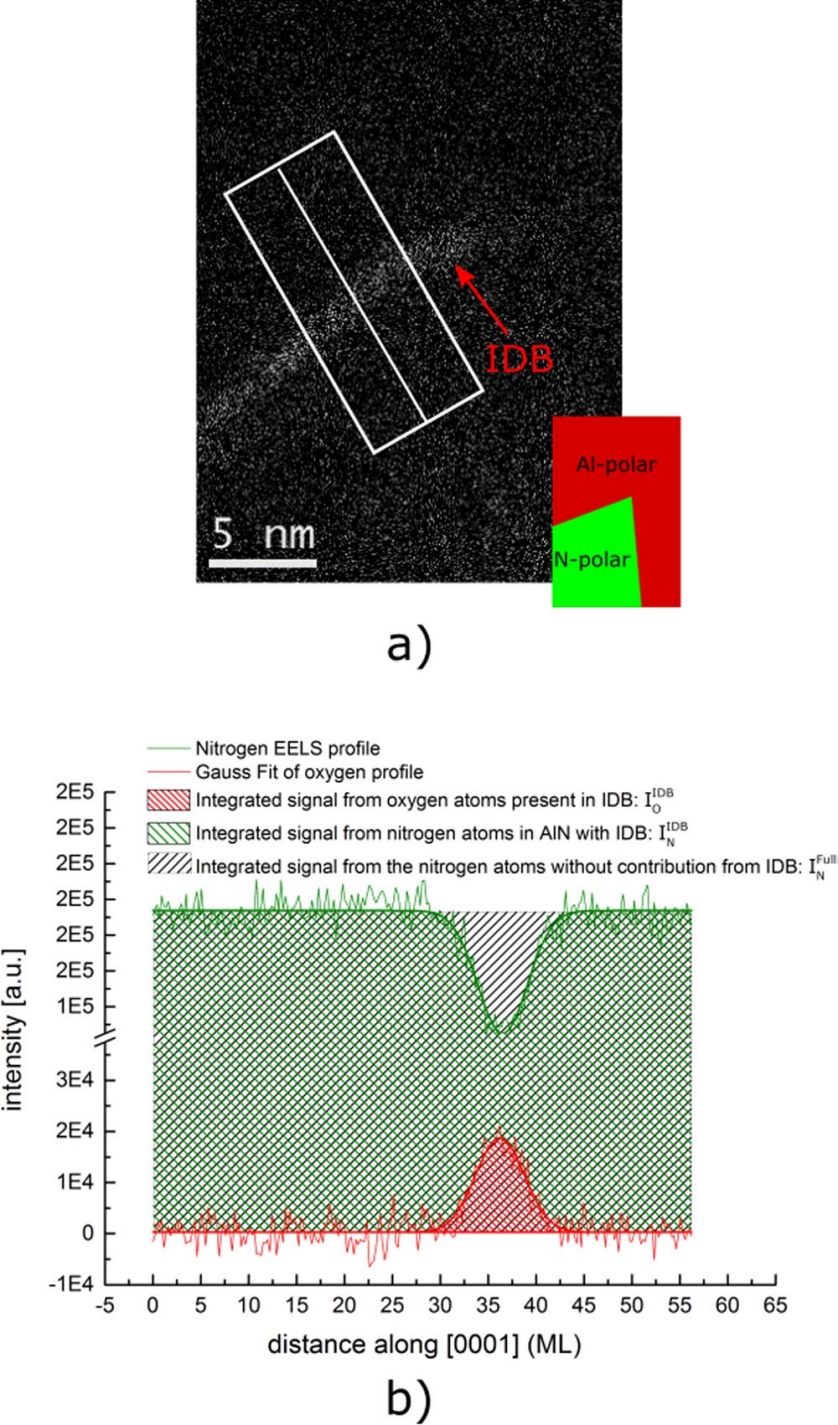
EELS analysis of the inversion domain boundary. (**a**) Map of oxygen K-edge integrated intensity within the area of IDB, schematically shown on the inset. (**b**) Integrated O and N K-edge signal profiles across the area with IDB and their Gaussian fittings. (The green shaded area corresponds to ${I}_{N}^{IDB}$ — integrated EELS intensity of N within the measured volume of AlN with IDB; the black shaded area (${I}_{N}^{Full}$) — the total integrated intensity of N that would be if IDB was not present within the measured volume; the red shaded area (${I}_{O}^{IDB}$) — the integrated EELS intensity of O within the measured volume of AlN with IDB).

To prove that the inversion domain boundary consists of Al_x_O_y_N_z_ and to quantify the oxygen content, we analyze it by spatially-resolved electron energy-loss spectroscopy and map the oxygen integrated intensity (Fig. 4(a)). In particular, we studied a region, in which the N-polar matrix and an Al-polar domain lie aside from each other. As described above, the N-polar domain switches its polarity, while the Al-polar domain does not (see schematic representation in the inset). The integrated line profile shows that oxygen is predominantly present within the lateral inversion boundary (where the N polar column switches to Al polarity) but is not measurable in the Al-polar columnar domain (i.e., where no polarity inversion occurs).

Figure 4(b) shows the integrated intensity profiles of the O and N K-edges across the IDB (along the white rectangular in Fig. 4(a)) versus distance converted to basal plane monolayers. The rise of oxygen signal within the IDB is correlated with a drop of the nitrogen signal within the same area — an indication that oxygen atoms partially substitute nitrogen atoms within the AlN wurtzite lattice, likewise in the Al_9_O_3_N_7_ structure mentioned above, where the oxygen and nitrogen atoms share the anion sites.

To estimate the oxygen content within the IDB, we utilize the equation relating the EELS integrated intensities and concentrations of two elements^29^:

1 $$\frac{{C}_{O}^{IDB}}{{C}_{N}^{IDB}}=\frac{{I}_{O}^{IDB}}{{I}_{N}^{IDB}}\times \frac{{\sigma }^{N}(\beta ,{\rm{\Delta }})}{{\sigma }^{O}(\beta ,{\rm{\Delta }})}=\frac{{I}_{O}^{IDB}}{{I}_{N}^{IDB}}\times {k}^{N,O}(\beta ,{\rm{\Delta }}),$$

where ${C}_{O,N}^{IDB}$ is the volume density of atoms for oxygen and nitrogen within the investigated volume; *I*^*O*,*N*^ is the intensity of the core-loss oxygen and nitrogen signals collected within the semi-angle β and integrated over an energy range of Δ; *σ*^*O*,*N*^(*β*, *Δ*) is a partial cross-section of O and N, calculated for core-loss scattering up to an angle β and energy beyond the edge onset of Δ.

We estimate the cross-section ratio *k*^*N*,*O*^ by calculating it near the Al_2_O_3_/AlN interface, where the nominal concentrations of O in Al_2_O_3_ and N in AlN are known. The nitrogen content ${C}_{N}^{IDB}$ is obtained from the ratio ${I}_{N}^{IDB}/{I}_{N}^{Full}$ (see Fig. 4(b)), where ${I}_{N}^{IDB}$ – integrated EELS intensity of N within the measured volume of AlN with the IDB (the green shaded area on the Fig. 4(b)), and ${I}_{N}^{Full}$ — the total integrated intensity of N that would be if the IDB was not present within the measured volume (the black shaded area on Fig. 4(b)).

Then, using the equation (1), where ${I}_{N}^{IDB}$ and ${I}_{O}^{IDB}$ are the integrated intensities of N and O across the boundary (Fig. 4(b)), we have estimated that the accumulated content of O atoms within the IDB ${C}_{O}^{IDB}$ yields an equivalent of 1.5 basal plane monolayers of the anion sub-lattice of the wurtzite structure.

## Discussion

Our investigation shows that the polarity of N-polar AlN films can be intentionally switched by an oxygen annealing as a mediating step in MOVPE growth. STEM and EELS experiments showed that oxygen atoms partially substitute nitrogen atoms in the N-polar wurtzite lattice, bond with Al-atoms, and form an Al_x_O_y_N_z_ inversion domain boundary, which inverts the polarity to Al. The structure qualitatively agrees with the Al_9_O_3_N_7_ model that forms during nitridation of the sapphire substrate and also promotes polarity inversion as proposed by Mohn *et al*.^22^. Al-polar surfaces, however, do not switch their polarity after oxygen treatment, and Al_x_O_y_N_z_ is not present. Thus, the AlN film, deposited after oxygen annealing, is predominantly Al-polar as opposed to the mainly N-polar AlN formed during the first growth step before annealing. The fact that we do not observe a conversion from Al polarity to N-polarity might be related to the differences in the oxygen incorporation into the different surfaces. Previous experimental and theoretical studies show that oxygen atoms more favorably adsorb on N-polar GaN and AlN surfaces than on metal polar ones since the adsorption energy at high oxygen coverage is significantly lower for the (000–1)-polar AlN and GaN surface compared to the (0001)-polar surface^1,30–33^.

**Figure 5:**
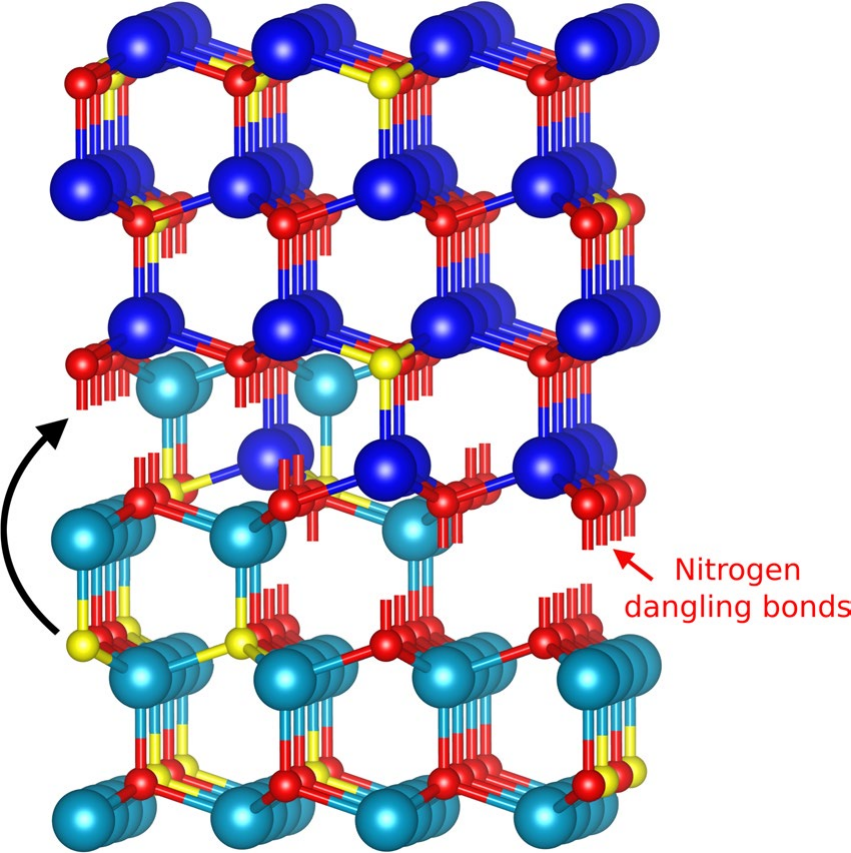
Schematic representation of charge compensation within inversion domain boundary between N-polar (light blue balls as Al cations) and Al-polar (dark blue balls as Al cations) interpenetrating lattices. Small red and yellow balls denote N and O atoms, respectively. The black arrow schematically indicates a transfer of electrons from an oxygen donor to N dangling bonds in the IDB.

It is interesting to note, that the total amount of oxygen incorporated into the interface between N-polar AlN and overgrown Al-polar is limited to an equivalent of 1.5 basal plane monolayers. This is precisely the amount found by Westwood *et al*.^34,35^ and Bruley *et al*.^36^, who studied the reverse inversion from metal polar to the N-polar surface. These authors explained this limitation by charge compensation necessary to fulfill the Pauling rule. A straightforward consideration may explain this similarity between our results: Above we describe the IDB by two interpenetrating wurtzite lattices that share a common anion sub-lattice. This means that at the inversion boundary each respective cation sub-lattice is terminated, providing one N dangling bond along the c-axis per 1 × 1 wurtzite interface area each (see Fig. 5). To saturate the dangling bond ¾ of an electron is required (in the wurtzite lattice Al atoms provide three valence electrons to the four Al-N bonds). Assuming that substitutional oxygen in the AlN lattice acts as a shallow donor and provides one free electron per incorporated oxygen atom, an amount of 1.5 oxygen atoms per 1 × 1 IDB interface area, i.e., 1.5 basal plane monolayers, would, therefore, lead to charge compensation.

## Conclusion

Summarizing, we have demonstrated a way to intentionally invert N-polar AlN layers to Al-polar by introducing annealing in an oxygen-rich environment into the MOVPE growth process. We showed that the oxygen annealing induces an inversion domain boundary consisting of a self-limited aluminum-oxynitride structure, which forms due to oxygen incorporation into N-polar surfaces and establishes Al-polarity of the film. Incorporation of an equivalent of 1.5 basal plane monolayers of oxygen is sufficient to enable this mechanism. This effect offers a method of controlled polarity inversion of N-polar films along the growth direction during MOVPE growth and realization of N-Al hetero-polar interfaces. Besides, the selective effect of oxygen incorporation on N-polar surfaces offers a way to suppress unwanted N-polarity in the growth of metal polar III-Nitride nanowires. It might also be used to control the size of N- and Al-polar domains in mixed-polar buffers, thus offering a method to reduce the dislocation density in AlN buffer layers for deep-UV emitters by a lateral overgrowth of domains with different polarities.

## Methods

### The growth of AlN film

The studied samples are grown in a vertical home-built MOVPE reactor. A mixture of H_2_ and N_2_ acts as a carrier gas. Trimethylaluminum and NH_3_ gas are used as Al and nitrogen sources, respectively. The flow rates of NH_3_, TMAl, H_2,_ and N_2_ are 3.5, 0.005, 5.0, and 3.0 slm, respectively. The resulting V/III ratio is approximately 130000. The epi-ready c-plane 2″ sapphire substrate is initially nitridated at atmospheric pressure for 30 minutes at 1080 °C in an NH_3_ gas flow. Then AlN film approximately 280 nm thick is deposited at the same temperature. The total pressure in the reactor during the AlN deposition is 300 mbar.

After the first step of AlN deposition, the process is interrupted, and the sample is transferred into a plasma-assisted molecular beam epitaxy (MBE) chamber. Inside the MBE chamber, the sample is heated up to around 550 °C for 30 minutes under a flow of 0.2 sccm of oxygen. RF power of 420 W is used to generate active oxygen. The pressure in the chamber is 10^−9^ Torr before introducing oxygen, reaches 10^−6^ Torr after 2 minutes of annealing under oxygen plasma and increases up to 6 × 10^−6^ Torr at the end of the annealing. After annealing in oxygen plasma, the sample is transferred back to the MOVPE growth reactor, where a 200 nm thick AlN film is grown on top of the oxygen-plasma treated sample surface at 1080 °C.

### TEM analysis

The samples for TEM investigations are prepared in cross-sectional geometry by mechanical polishing (diamond foils) down to a sample thickness of 8–10 µm. The samples are then thinned to electron transparency by argon ion milling at accelerating voltages decreasing stepwise from 4 to 0.2 kV at an angle of 4° in a GATAN precision ion polishing system (PIPS).

Conventional TEM investigations are done in an FEI Titan 80–300 operating at 300 kV. High-resolution HAADF-STEM and EELS investigations are performed with a NION Ultrastem microscope operating at 200 kV with a cold field emission gun as an electron source and a probe correction system, providing a spatial resolution of 0.7 Å and an energy resolution of EELS measurements of 0.28 eV. The values of convergence and EELS collection semi-angles remained constant for each experiment and are 28 mrad and 24 mrad, respectively. EELS measurement are performed in a fast acquisition mode (171.15 spectra/sec) and low probe current (50–80 pA) conditions. This mode allows suppressing the effect of strong radiation damage of the specimen while maintaining high spatial resolution, at the expense of a low signal-to-noise ratio. The EELS data evaluation is performed with the Gatan Microscopy Suite software (ver. 3.11). For compositional analysis, the core-loss intensity is integrated over an energy range of 50 eV beyond the edge onset, which leads to averaging the contribution of the near-edge structure.

## Data Availability

The datasets generated and analyzed during the current study are available from the corresponding author on reasonable request.
